# Assessing Bioavailability and Bioactivity of 4-Hydroxythiazolidine-2-Thiones, Newly Discovered Glucosinolate Degradation Products Formed During Domestic Boiling of Cabbage

**DOI:** 10.3389/fnut.2022.941286

**Published:** 2022-07-22

**Authors:** Holger Hoffmann, Christiane Ott, Jana Raupbach, Lars Andernach, Matthias Renz, Tilman Grune, Franziska S. Hanschen

**Affiliations:** ^1^Plant Quality and Food Security, Leibniz Institute of Vegetable and Ornamental Crops (IGZ), Großbeeren, Germany; ^2^Department of Molecular Toxicology, German Institute of Human Nutrition Potsdam-Rehbruecke (DIfE), Nuthetal, Germany; ^3^Institute of Nutritional Science, University of Potsdam, Nuthetal, Germany; ^4^Department of Physiological Chemistry, Faculty of Chemistry, University of Vienna, Vienna, Austria

**Keywords:** stomach model, glycogen synthase kinase-3, cytotoxicity, antioxidant potential, intestinal model, cellular uptake, isothiocyanate

## Abstract

Glucosinolates are plant secondary metabolites found in cruciferous vegetables (Brassicaceae) that are valued for their potential health benefits. Frequently consumed representatives of these vegetables, for example, are white or red cabbage, which are typically boiled before consumption. Recently, 3-alk(en)yl-4-hydroxythiazolidine-2-thiones were identified as a class of thermal glucosinolate degradation products that are formed during the boiling of cabbage. Since these newly discovered compounds are frequently consumed, this raises questions about their potential uptake and their possible bioactive functions. Therefore, 3-allyl-4-hydroxythiazolidine-2-thione (allyl HTT) and 4-hydroxy-3-(4-(methylsulfinyl) butyl)thiazolidine-2-thione (4-MSOB HTT) as degradation products of the respective glucosinolates sinigrin and glucoraphanin were investigated. After consumption of boiled red cabbage broth, recoveries of consumed amounts of the degradation products in urine collected for 24 h were 18 ± 5% for allyl HTT and 21 ± 4% for 4-MSOB HTT (mean ± SD, *n* = 3). To investigate the stability of the degradation products during uptake and to elucidate the uptake mechanism, both an *in vitro* stomach and an *in vitro* intestinal model were applied. The results indicate that the uptake of allyl HTT and 4-MSOB HTT occurs by passive diffusion. Both compounds show no acute cell toxicity, no antioxidant potential, and no change in NAD(P)H dehydrogenase quinone 1 (NQO1) activity up to 100 μM. However, inhibition of glycogen synthase kinases-3 (GSK-3) in the range of 20% for allyl HTT for the isoform GSK-3β and 29% for 4-MSOB HTT for the isoform GSK-3α at a concentration of 100 μM was found. Neither health-promoting nor toxic effects of 3-alk(en)yl-4-hydroxythiazolidine-2-thiones were found in the four tested assays carried out in this study, which contrasts with the properties of other glucosinolate degradation products, such as isothiocyanates.

## Introduction

Vegetables from the family Brassicaceae, “the cabbage and mustard family,” naturally contain metabolites known as glucosinolates (GLSs). Many of these vegetables belong to the botanical genus *Brassica*, including cauliflower, Brussels sprouts, radish, cabbage, and broccoli. Consumption of these vegetables contributed the most to the total intake of GLSs with an average daily intake of 14–15 mg GLSs in Germany (1). Pfaff et al. (2) reported that the average daily intake was found to be 54 g *Brassica* with cauliflower, red cabbage, and white cabbage as the predominant species. High dietary intake of GLS-containing *Brassica* vegetables correlates with decreased cancer risks such as lung, stomach, and colon cancer (3). However, this effect is mostly based on the degradation products of GLSs, such as isothiocyanates (ITCs). These can be formed during enzymatic hydrolysis of GLSs by myrosinase to an unstable thiohydroximate-*O*-sulfate, which reacts to the ITC by a Lossen-like rearrangement (4). ITCs are linked to several health-promoting effects like anticancerogenic activities, including modulation of phase I and II enzymes, inhibition of cell growth, prevention of metastasis, and regulation of epigenetic machinery (5, 6). Additionally, ITCs have antimicrobial (7, 8), antifungal (9–11), and antiherbivorous properties (12, 13). Approximately, between 88 and 137 GLSs are considered to be identified from Brassicales plants (14). Among them, the GLSs glucoraphanin (4-(methylsulfinyl)butyl GLS) and sinigrin (allyl GLS) are well-known due to their occurrence and the biological effects of their enzymatic degradation products sulforaphane and allyl isothiocyanate (allyl ITC). Sulforaphane is one of the most bioactive GLS hydrolysis products (15) and showed chemopreventive and anticancer effects by up- and down-regulation of several pathways (16). Likewise, in several studies, similar activities were reported for allyl ITC (17).

Regarding the fact that many *Brassica* vegetables are frequently boiled before consumption, degradation of GLSs and ITCs should be considered. Depending on pH value and boiling time, ITCs can react to thiourea derivatives during aqueous heating (18). Moreover, GLSs such as sinigrin can be degraded to nitriles and thioglucose during heat treatment (19). Recently, we identified 3-alk(en)yl-4-hydroxythiazolidine-2-thiones (Alk(en)yl HTTs) as a class of GLS degradation products, which were formed during aqueous heating by the reaction of ITCs and mercaptoacetaldehyde derived from thioglucose as a thermal degradation product of GLSs (20). It was shown that 3-allyl-4-hydroxythiazolidine-2-thione (allyl HTT), the reaction product of allyl ITC and mercaptoacetaldehyde, and 4-hydroxy-3-(4-(methylsulfinyl)butyl)thiazolidine-2-thione (4-MSOB HTT), as a product of sulforaphane and mercaptoacetaldehyde, were detected in boiled cabbage samples. The consumed amount of the novel degradation products was estimated to be in the nanomolar range for an ordinary meal. This study aimed to evaluate the potential of these HTTs as thermal GLS degradation products to (a) be taken up in the human circulatory system and (b) act as bioactive compounds. The recently calculated log *P* (partition coefficient in octanol/water) values of 0.47 and −1.12 for the two newly discovered compounds allyl HTT and 4-MSOB HTT (20) indicate whether the compounds have the potential to be taken up by cells through passive diffusion. The optimal log *P* range for intestinal absorption is suggested between 0.5 and 2 (21). Regarding bioactivity, it should be noticed that the reactive ITC group is no longer present in the HTTs, resulting in higher stability of the newly discovered degradation products compared to the corresponding ITCs, but, likely, also in a lower potential to act as a health-promoting compound. However, structurally similar compounds to the HTTs, such as 3-thiazolidine-4-ones, have a wide range of activities, such as antimicrobial, anti-inflammatory, or anticancer properties (22). Allyl ITC has effects on the transcription factor nuclear factor erythroid 2-related factor 2 (Nrf2) (23). Therefore, the acute cytotoxicity and the potential of the thermal GLS degradation products allyl HTT and 4-MSOB HTT to act as bioactive compounds and to interact with transcription factor Nrf2 were investigated. As the inhibition of glycogen synthase kinases-3 (GSK-3) by structurally similar compounds as the HTTs was shown by Noori et al. (24), their ability to inhibit GSK-3, which is involved in the regulation of Nrf2, was examined.

## Materials and Methods

### Chemicals

**Figure 1:**
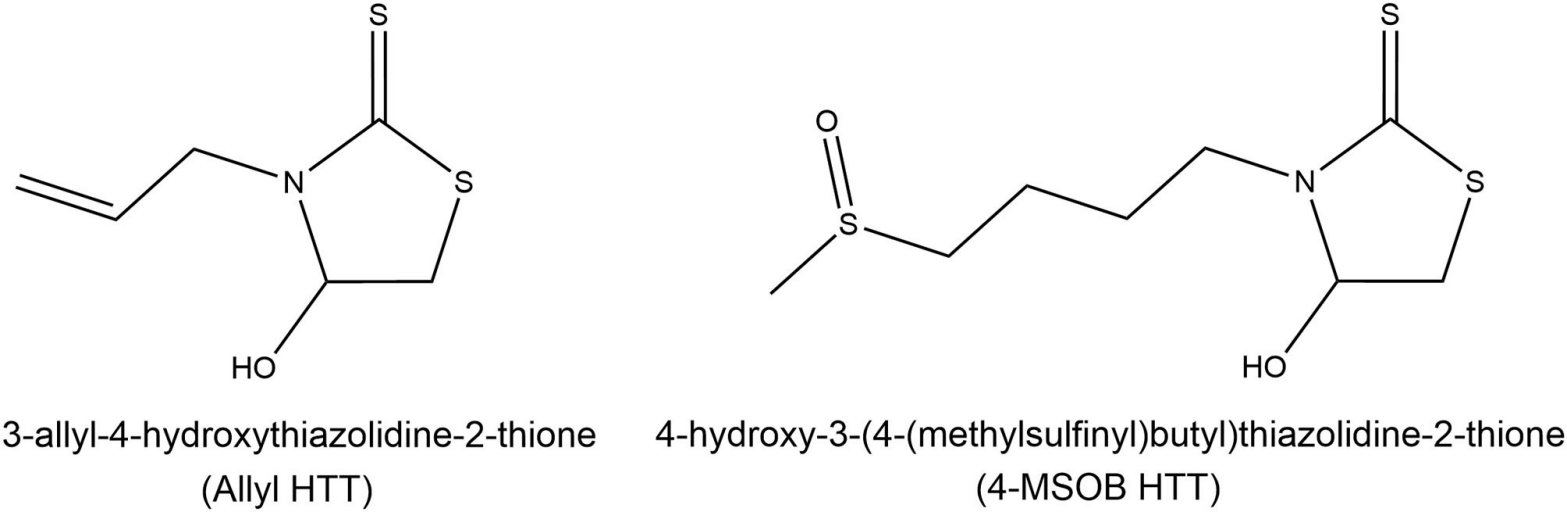
Structures of the recently discovered heat-induced glucosinolate degradation products 3-allyl-4-hydroxythiazolidine-2-thione (allyl HTT, left) and 4-hydroxy-3-(4-(methylsulfinyl)butyl)thiazolidine-2-thione (4-MSOB HTT, right). Modified according to Hoffmann et al. (20).

The thermal GLS degradation products allyl HTT and 4-MSOB HTT were synthesized as described by Hoffmann et al. (20). Their structures are shown in Figure 1. ABTS^TM^ (2,2′-azino-bis(3-ethylbenzothiazoline-6-sulfonic acid) diammonium salt, >98%) was purchased from Roche (Mannheim, Germany). Additionally used compounds were 1-thio-β-d-glucopyranosatotriethyl phosphine gold-2,3,4,6-tetraacetate (Auronafin, ≥98%) from Enzo Life Sciences (Lörrach, Germany), McCoy's 5A Medium (1x) gibco (+ l-glutamine), GlutaMAX^TM^, and hygromycin B from Thermo Fisher Scientific GmbH (Dreieich, Germany), as well as penicillin/streptomycin (100 x, 10,000 U/ml penicillin G, 10 mg/ml streptomycin, sterile filtered) from GENAXXON bioscience (Ulm, Germany). For cell culture experiments, gibco's Dulbecco's Modified Eagle Medium (DMEM) (1x) (+ 4.5 g/L d-glucose, − sodium pyruvate), RPMI-1640 medium (l-glutamine, sodium bicarbonate, sterile filtered, for cell culture), trypsin/EDTA (10 x), and heat-inactivated fetal bovine serum (FBS) were purchased from Bio&Sell (Feucht/Nürnberg, Germany). KCl (>99.5%, p.a.), KH_2_PO_4_ (>99.5%, p.a.), 3-(4,5-dimethylthiazol-2-yl)-2,5-diphenyltetrazolium bromide (MTT), 5% formic acid in isopropanol, and 3-amino-7-dimethylamino-2-methylphenazine hydrochloride (neutral red, powder, BioReagent, suitable for cell culture) were purchased from Merck KGaA (Darmstadt, Germany). 1-Octanol (≥99.5%,), NaCl (≥99.8% with anti-caking agent), dimethyl sulfoxide (DMSO, ≥99.5%, ROTIDRY^®^), HCl (ROTIPURAN^®^ 37%, p.a., ACS, ISO), acetic acid (ROTIPURAN^®^ 100%, p.a.), and phosphate-buffered saline (PBS) (137 mM NaCl, 2.7 mM KCl, 1.5 mM KH_2_PO_4_, 8.1 mM Na_2_HPO_4_·2 H_2_O, pH 7.4) were all procured from Carl Roth + Co. KG (Karlsruhe, Germany). Pepsin (≥ 3,200 U/mg, from porcine gastric mucosa), mucin-type II (from the porcine stomach), Trolox^®^ [(±)-6-hydroxy-2,5,7,8-tetramethylchromane-2-carboxylic acid, 97%], potassium peroxodisulfate (≥99%, puriss p.a. ACS reagent), and DPPH (2,2-diphenyl-1-picrylhydrazyl) were all obtained from Sigma-Aldrich (Steinheim, Germany). For LC-MS measurements, water (LiChrosolv^®^, LC-MS grade) from Merck KGaA (Darmstadt, Germany) and an organic solvent or mobile phase for HPLC measurements containing acetonitrile (MeCN, CHEMSOLUTE^®^, >99.95%, LC-MS grade) and methanol (MeOH, CHEMSOLUTE^®^ ≥99.95%, for LC-MS) purchased from Th. Geyer GmbH and Co. KG (Renningen, Germany) were used.

### Bioavailability of 4-Hydroxythiazolidine-2-Thiones

#### Uptake and Excretion of 4-Hydroxythiazolidine-2-Thiones From a Red Cabbage Meal – A Human Self-Experiment

To obtain the GLS degradation products allyl HTT and 4-MSOB HTT in the food matrix, a kitchen practice approach was applied. Therefore, 1.2 kg of fresh conventional red cabbage (*Brassica oleracea* var. *capitata* f. *rubra*) bought in a supermarket were cut into approximately 4 × 4 × 4 cm pieces and boiled for 1 h in 3 L of tap water without additives in a stainless-steel pot with a glass lid on a kitchen stove. After cooling to room temperature, three male authors of this study (27–39 years) drank 500 mL of the red cabbage boiling solution (broth) without red cabbage pieces. After a 72 h wash-out phase with no consumption of food products containing GLSs and shortly before consumption of the red cabbage broth, 40 mL of urine were collected to test the absence of allyl HTT and 4-MSOB HTT. After consumption, urine was collected for 24 h in a urine container (3 L, Sarstedt AG & Co, Nümbrecht, Germany). For allyl HTT, adsorption effects on the polyethylene surface were detectable. Therefore, the urine was transferred to glassware, and the urine containers were washed 3 times with 200 mL of MeCN and the obtained MeCN and urine were combined. Determination of allyl HTT and 4-MSOB HTT in urine and the red cabbage solution was performed by using LC-MS/MS. An HPLC-system from Agilent Technologies Germany (Waldbronn) with Agilent 1260 Infinity: (binary pump, autosampler, degasser, column oven, DAD detector) was used, with an Agilent Poroshell 120 column (PFP 2.1 × 100 mm, 2.7 μm), coupled with an AB Sciex 6500 QTRAP mass spectrometer (Sciex, Darmstadt, Germany). Red cabbage boiling solution was diluted 1:1 with water and centrifuged for 3 min at 4,000 *g*. The standard addition method was used to determine absolute amounts of analyte in the samples. In total, 950 μL of 1:1 diluted red cabbage boiling solution was measured in 3 different ways (each prepared as duplicates): (1) Spiked with 50 μL water, (2) spiked with 50 μL of a solution with concentrations of 1 μM for allyl HTT and 5 μM for 4-MSOB HTT, or (3) spiked with 50 μL of a solution with concentrations of 3 μM for allyl HTT and 10 μM for 4-MSOB HTT. Urine samples were measured in three different ways (each prepared as duplicates): 200 μL of undiluted urine was spiked with (1) 1 μL of water, (2) 1 μL of a solution with concentrations of 4 μM for allyl HTT and 8 μM for 4-MSOB HTT, or (3) 1.5 μL of a solution with concentrations of 4 μM for allyl HTT and 8 μM for 4-MSOB HTT. The injection volume was 40 μL. Mass spectrometry parameters (QTRAP method 1) were ESI positive, TEM 500°C, IS 5500 V, CUR 50 psi, GS1 50 psi, and GS2 60 psi with MS/MS parameters are given in Table 1. The used HPLC method 1 was a gradient of A = water and B = MeCN with 0 to 5 min 2% B, an increase to 14 min up to 98% B, holding for 2 min followed by a decrease to 2% B at 17 min and re-equilibration with 2% B for 4 min, using a flow rate of 300 μL/min and a column oven temperature of 35°C. The results are reported as the mean of the three biological replicates ± the standard deviation (SD).

**Table 1:** Mass transition parameters for determination of 3-allyl-4-hydroxythiazolidine-2-thione (allyl HTT) and 4-hydroxy-3-(4-(methylsulfinyl)butyl)thiazolidine-2-thione (4-MSOB HTT) by LC-MS/MS.

**Compound species**	**Mass transition**	**CE (V)**	**DP (V)**	**EP (V)**	**CXP (V)**
[Allyl HTT+H]^+^	176 → 100 (quantifier)	17	30	10	11
	176 → 75 (qualifier)	17	30	10	11
	176 → 67 (qualifier)	25	30	10	11
	176 → 41 (qualifier)	45	30	10	11
-MSOB HTT+Na^+^	276 → 182 (quantifier)	22	60	15	11
	276 → 258 (qualifier)	20	60	15	11
	276 → 212 (qualifier)	17	60	15	11
	276 → 200 (qualifier)	22	60	15	11

#### Stability of 4-Hydroxythiazolidine-2-Thiones in a Stomach Model

To investigate the stability of the HTTs in the process of nutrient absorption, allyl HTT and 4-MSOB HTT were incubated (prepared as triplicates) at a concentration of 100 μM in an *in vitro* stomach model solution containing 2.9 g/L NaCl, 0.7 g/L KCl, 0.27 g/L KH_2_PO_4_, 0.78 g/L pepsin, and 3 g/L mucin-type II and adjusted with 1 M HCl to pH 2.0 based on literature (25). To simulate the stomach, samples were shaken slightly for 2 h at 200 rpm in a thermoshaker (MHR 23, Hettich Benelux B.V, Netherlands) at 37°C. Afterward, 500 μL of MeCN were added to 500 μL of stomach model solution to precipitate proteins and salts. The suspension was centrifuged for 3 min at 4,000 *g* and the supernatant was analyzed by HPLC-UV at a wavelength of 274 ± 2 nm. The stability of allyl HTT and 4-MSOB HTT in the stomach model was determined by the recovery compared to incubation in pure water at room temperature. The UPLC-DAD system was from Agilent Technologies Germany (Waldbronn) (Agilent 1290 Infinity: (binary pump, autosampler, degasser, and column oven) and Agilent 1260 Infinity: (DAD and FLD detectors) with an injection volume of 10 μL. As for column, Agilent Zorbax Eclipse Plus C8 (RRHD 3 × 100 mm, 1.8 μm) with Agilent pre-column EC-C18 (2.1 × 5 mm, 2.7 μm) was used. The applied HPLC method 2 was a gradient of A = water and B = MeCN with 0 to 5.1 min 2% B, increasing to 98% B at 14 min, holding 98% B for 5.4 min, followed by decreasing within 0.6 min to 2% B and re-equilibrating at 2% B for 4.5 min (24.5 min), using a flow rate of 300 μL/min and a column oven temperature of 35°C. The reported recoveries are the mean ± SD of the obtained results.

#### Uptake of 4-Hydroxythiazolidine-2-Thiones Into HepG2 Cells

Cells originated from a human liver cancer cell line (HepG2) were grown in RPMI-1640 medium + 10% FBS + 1% penicillin/streptomycin under cell culture conditions (37°C and 5% CO_2_ atmosphere) in T-75 flasks (Sarsted, Nümbrecht, Germany) until a confluence of approximately 50%. This medium and growing conditions were used for all experiments with HepG2 cells in this study. The medium was exchanged by fresh medium for control samples, or by fresh medium containing a concentration of 100 μM allyl HTT or 4-MSOB HTT. Afterward, the cells were incubated for an additional 24 h (each prepared for three cell passages). In the case of cell experiments, all allyl HTT or 4-MSOB HTT spiked media were sterile filtered using Filtropur S 0.2 sterile from Sarstedt AG & Co (Nümbrecht, Germany). After treatment, the medium of HepG2 cells (~80% confluence) was removed and cells were washed three times with 6 mL of PBS. In a second approach performed only for 4-MSOB HTT, treated cells were washed 6 times with 6 mL of PBS. The higher number of washing steps in the case of 4-MSOB HTT was chosen to remove the analyte residues from the former treatment medium more effectively. This enabled to detect a significant increase in analyte amount due to cell lysis when comparing the analyte amount in the supernatant before and after cell lysis, as only three washing steps for allyl HTT and control were not sufficient to detect a significant difference in 4-MSOB HTT amounts due to cell lysis treatment. Afterward, cells were detached from the surface by incubation with 1.5 mL trypsin/EDTA (1 x) for 5 min at 37°C. The cell suspension was transferred into a 2-mL micro reaction vessel and centrifuged for 2 min at 560 *g*. Then, the cell pellet was washed carefully twice with 1.5 mL of pure water to remove further residues of the analyte spiked medium and to reduce the salt concentration in the samples for LC-MS/MS measurements. For cell lysis, the micro reaction vessels with cell pellet and approximately 60 μL supernatant were sonicated for 15 min in an ultrasonic bath, followed by the addition of 40 μL of MeCN and an additional sonication step for 15 min. Afterward, the cell suspension was centrifuged for 3 min at 4,000 *g*, and the supernatant, as well as the supernatant before cell lysis, were analyzed by LC-MS/MS. A standard addition method was used, with added amounts of 3 pmol of allyl HTT and 1.2 pmol of 4-MSOB HTT. The mass spectrometry parameters were like QTRAP method 1 (see Section Uptake and Excretion of 4-Hydroxythiazolidine-2-Thiones From a Red Cabbage Meal – A Human Self-Experiment) with an injection volume of 10 μL. The used HPLC method 3 was a gradient of A = water and B = MeCN with 0 to 5 min 2% B, an increase to 14 min up to 98% B, holding for 5 min followed by a decrease to 2% B at 20 min and re-equilibration with 2% B for 4 min, using a flow rate of 300 μL/min, Agilent Zorbax Eclipse Plus C8 column (see Section Stability of 4-Hydroxythiazolidine-2-Thiones in a Stomach Model) and a column oven temperature of 35°C. The given results are the mean of the three biological replicates ± SD. The difference between before and after cell lysis was calculated for each biological replicate separately and based on these results the mean ± SD was calculated. The significance of the difference in analyte amount before and after cell lysis was determined by *t*-test for independent samples per variable using the STATISTICA version 13.5 software (StatSoft, Hamburg, Germany) with a significance level of *p* ≤ 0.05.

Additionally, the removed non-spiked and spiked medium after 24 h of cell incubation was measured by the UPLC-DAD system (see Section Stability of 4-Hydroxythiazolidine-2-Thiones in a Stomach Model) and HPLC method 2 at 280 ± 2 nm and after 10-fold pre-concentration, using solid-phase extraction (Chromabond^®^, C18, 3 mL/500 mg, Macherey-Nagel) by an HPLC-DAD-ESI-qToF system (Agilent 1290 Infinity II for the binary pump, Agilent 1290 Infinity for autosampler, degasser, column oven, Agilent 1260 Infinity for DAD detector, and Agilent 1260 Infinity II, for an isocratic pump for adding reference mass solution) using A = water and B = MeCN: 0 to 3 min 2% B, an increase to 14 min up to 98% B, holding for 5.4 min followed by a decrease to 2% B at 20 min, and re-equilibration with 2% B for 4.5 min, using a flow rate of 300 μL/min, Agilent Zorbax Eclipse Plus C8 column (see Section Stability of 4-Hydroxythiazolidine-2-Thiones in a Stomach Model), and a column oven temperature of 35°C. The Agilent ESI-qToF (G6546A) with Dual AJS ESI source operating in positive ionization mode with VCap 3500 V, nozzle voltage 0 V, fragmentor 175 V, skimmer 1 65 V, octopoleRFPeak 750 V, mass range *m/z* 100–950, gas temperature 200°C, gas flow 8 L/min, nebulizer 35 psig, sheath gas temperature 350°C, sheath gas flow 12 L/min, and using reference mass solution Agilent HP 921 with purine (reference *m/z* 121.0509 and *m/z* 922.0098).

#### Transport of 4-Hydroxythiazolidine-2-Thiones Through *in vitro* Model of the Intestinal Barrier

Human colorectal adenocarcinoma 2 (Caco-2) cells were grown in DMEM medium + 10% FBS + 1% penicillin/streptomycin under cell culture conditions (37°C and 5% CO_2_ atmosphere). This medium and growing conditions were used for all experiments with Caco-2 cells. For modeling the intestinal barrier, Caco-2 cells were seeded in transwell inserts ThinCert^TM^ – TC inserts (6 well, 3 μm, sterile) from Greiner Bio-One with a seeding density of 100,000 cells/cm^2^ based on the work of Hurley et al. (26). To obtain a closed differentiated Caco-2 cell layer, a proliferation time of 21 days, with medium exchange every 2–3 days, was chosen. The integrity of the cellular barriers was determined by transepithelial electrical resistance (TEER) measurement (model EVOM-G, World Precision Instruments with STX2 electrode) at different time points during the proliferation time, and at the end of analyte treatment and sample collection. After 21 days, the medium was exchanged for fresh medium for control samples, or by fresh medium containing a concentration of 100 μM allyl HTT or 4-MSOB HTT, each prepared for 6 cell passages. In the first experiment, the HTT was added to the upper compartment (in the transwell insert above the cell layer) and in the second experiment as an inverse exposure in the lower compartment (in the well below the cell layer). The added volume was 2 mL medium in the transwell insert (upper compartment) and 2.7 mL medium in the well (lower compartment) to obtain a hydrodynamic equilibrium (Supplementary Figure S1). After an incubation time of 2 min, 1 h, 2 h, and 24 h under cell culture conditions, 200 μL of the upper compartment and 270 μL of the lower compartment were taken and measured by HPLC-UV at 274 ± 2 nm using the HPLC method 2 (see Section Stability of 4-Hydroxythiazolidine-2-Thiones in a Stomach Model) and an injection volume of 20 μL. The different volumes of sampling taken from the different compartments were chosen to maintain the hydrodynamic equilibrium. The determination of the HTT concentrations was performed using external calibration and is reported as the mean ± SD of the six biological replicates.

#### Determination of Log P (Partition Coefficient in Octanol/Water) Values of 4-Hydroxythiazolidine-2-Thiones

For the determination of log *P* of the HTTs, two solutions were used: water saturated with octanol and octanol saturated with water. Afterward, 10 μL of an aqueous solution with a concentration of 2 mM allyl HTT or 4-MSOB HTT was added to 190 μL of pure water using as reference, and for determination of the log *P*-value to a mixture of 90 μL of saturated water and 100 μL of saturated octanol (prepared as triplicates). The spiked octanol-water mixture was vortexed for 30 s and the mixture was incubated for 18 h at room temperature to obtain analyte equilibrium distribution. For determination of the log *P*-value, 50 μL of the water phase was taken and measured by HPLC-UV at 274 ± 2 nm with HPLC method 2 (see Section Stability of 4-Hydroxythiazolidine-2-Thiones in a Stomach Model) with an injection volume of 10 μL. The analyte concentration in the water phase was determined by using external calibration. The relative analyte content in the water phase was calculated by using the following equation:


$$\begin{array}{l} analyte\;content\;in\;water\;phase\;in\;\%\\ \;=\frac{measured\;concentration\;in\;water\;phase\;of\;the\;mixture}{2\;\cdot \;concentration\;in\;pure\;water\;reference}\;\cdot 100\% \end{array}$$

The relative analyte content in the octanol phase was calculated at 100% minus the relative analyte concentration in the water phase. The reported results are the mean ± SD of the three determined log *P*-values for the three repetitions.

### Bioactivity of 4-Hydroxythiazolidine-2-Thiones

#### Determination of Cytotoxic Effects of 4-Hydroxythiazolidine-2-Thiones

To determine the cytotoxicity of allyl HTT and 4-MSOB HTT, HepG2 and Caco-2 cells were seeded in a 96-well TC-plate (standard F, sterile, from Sarsted, Nümbrecht, Germany) using a seeding density of 20,000 cells/well. After 24 h, the medium was exchanged to 200 μL pure medium or 200 μL medium containing allyl HTT or 4-MSOB HTT with concentrations in the range of 0.1–100 μM. The cells were treated for 24 h under cell culture conditions (prepared for three biological replicates, every 3 technical replicates). Cell viability was determined using the MTT assay and the neutral red uptake assay based on Schröter et al. (27). In the case of MTT assay, after 24 h of HTT treatment, 20 μL of MTT solution (75 mg MTT in 15 mL PBS) were added, and cells were incubated for 60 min under cell culture conditions. Afterward, the supernatant was removed and 100 μL of lysis buffer (5% formic acid in isopropanol) was added and the plate was shaken for 10 min at 300 rpm before measuring the absorption at 550 ± 5 nm (reference 690 ± 5 nm) by a Tecan i-control Infinite M200 PRO plate reader (Crailsheim, Germany). In the case of the neutral red uptake assay, the supernatant was removed after 24 h HTT treatment, and 100 μL neutral red solution (40 mg neutral red in 10 mL PBS, freshly diluted 1:100 with medium and incubated for 24 h under cell culture conditions) was added. After incubation of neutral red solution for 2 h under cell culture conditions, the supernatant was removed, and cells were washed with 150 μL of PBS. After removing PBS, 150 μL desorb solution (49.5% water/49.5% ethanol/1% acetic acid) was added and the plate was shaken for 10 min at 300 rpm before measuring the absorption at 540 ± 5 nm by Tecan i-control Infinite M200 PRO plate reader. The mean ± SD is used for presenting the results of three technical replicates for each data point of the three biological replicates.

#### Determination of Antioxidant Potential of 4-Hydroxythiazolidine-2-Thiones

To evaluate the potential of allyl HTT and 4-MSOB HTT to act as antioxidants, ABTS and DPPH were used as model radicals and Trolox as reference antioxidants based on Shalaby and Shanab (28). In total, 1.5 mg of ABTS and 0.26 mg potassium peroxodisulfate were dissolved in 4 mL of pure water and incubated in the dark overnight at room temperature. Afterward, 36 mL of MeOH were added to obtain the fresh ABTS working solution. In the case of the DPPH assay, 1.1 mg of DPPH were dissolved in 4 mL of MeOH and incubated for 2 h in the dark at 4°C, followed by the addition of 36 mL of MeOH to obtain the fresh DPPH working solution. Standards of Trolox, allyl HTT, and 4-MSOB HTT were prepared in water/MeOH (90/10, v/v%) at concentrations in the range of 6.25–100 μM. To detect the antioxidant potential, 100 μL of standard or water/MeOH (90/10, v/v%) as control were added to 1,000 μL of ABTS or DPPH working solution, and the extinction was measured directly after mixing and after 6 min of incubation in the dark with a spectrometer (UviLine 9100, Schott Instruments, Mainz, Germany) at 734 nm in the case of ABTS assay and 515 nm in DPPH assay (each prepared as duplicate). The results are reported as mean ± SD of the reduction of the extinction compared to the control.

#### Determination of Glycogen Synthase Kinases-3 Inhibition by 4-Hydroxythiazolidine-2-Thiones

The determination of the inhibition of glycogen synthase kinases-3α and -3β by allyl HTT and 4-MSOB HTT was performed *via* contract research with Life Technologies/Thermo Fisher Scientific by sending the compounds dissolved in 100% DMSO to SelectScreen Services (Madison, WI, USA). The tested concentrations in the Z'-LYTE assay were up to 100 μM and standards were prepared as duplicates. The Z'-LYTE kinase assay is a fluorescence-based assay using a coupled-enzyme format and the different sensitivity of phosphorylated and non-phosphorylated peptides regarding their fluorescence. The data are shown as mean ± SD. Due to the impression of significant bioactivity, further comparisons were performed. To investigate if GSK-3 activity was significantly inhibited, values for each concentration level were compared to the concentration level of 0.14 μM (due to a lack of negative control) using the *t-*test for independent samples per variable using STATISTICA version 13.5 software (StatSoft, Hamburg, Germany) with a significance level of *p* ≤ 0.05. Stars report different significance levels: ^*^p ≤ 0.05, ^**^p ≤ 0.01, and ^***^p ≤ 0.001.

#### Determination of Changes in NAD(P)H Dehydrogenase Quinone 1 Activity by 4-Hydroxythiazolidine-2-Thiones

The effect of allyl HTT or 4-MSOB HTT on NAD(P)H dehydrogenase quinone 1 (NQO1) activity was tested for HepG2 cells and HCT116 pTRAF cells. HCT116 pTRAF is a cell line that was transfected with the pTRAF^Nrf2/HIF/NFκ*B*^ [Johansson et al. (29)], as described by Kipp et al. (30). HCT116 pTRAF cells were cultivated in McCoy's 5A medium + 1% penicillin/streptomycin + 10% FBS + 1% GlutaMAX + 0.2 mg/mL hygromycin B. HepG2 cells in RPMI medium were seeded in a 6-well-plate (sterile, Sarsted, Nümbrecht, Germany) with a density of 350,000 cells/well, and HCT116 pTRAF cells in culture medium without hygromycin B with a density of 100,000 cells/well. After 48 h of incubation under cell culture conditions, the medium was exchanged with the fresh medium as a negative control and a medium with concentrations of 1 μM and 100 μM of allyl HTT or 4-MSOB HTT. Medium containing 2 μM auranofin was used as a positive control. After 24 h of incubation under cell culture conditions, NQO1 activity was analyzed according to Müller et al. (31), and the results were reported as mean ± SD. Additionally, the *t*-test for independent samples per variable was performed to test if concentration-dependent differences exist.

## Results

### Bioavailability of 4-Hydroxythiazolidine-2-Thiones

#### Uptake and Excretion of 4-Hydroxythiazolidine-2-Thiones From a Red Cabbage Meal – A Human Self-Experiment

To test if the dietary compounds are taken up *in vivo*, a self-experiment was conducted. In the red cabbage broth that was prepared for the evaluation of uptake of the HTTs, 50 ± 10 nmol/L for allyl HTT and 252 ± 20 nmol/L for 4-MSOB HTT were quantified. Therefore, the amount taken up by drinking 500 mL of the broth was 25 ± 5 nmol of allyl HTT and 126 ± 10 nmol of 4-MSOB HTT. Both HTTs were not detected in urine samples before consumption of the red cabbage broth, after a 72 h wash-out phase for all three self-experimenting authors. To calculate the recovery of the excreted HTTs in urine compared to intake, the concentrations measured in urine were multiplied by the collected urine volumes to obtain absolute amounts of excreted compounds. Allyl HTT was excreted in the range of 3.5 ± 0.3 nmol up to 5.3 ± 0.8 nmol and 4-MSOB HTT in the range of 21.03 ± 0.03 nmol up to 31.6 ± 4.0 nmol for the three male experimenting authors. Therefore, recovery of allyl HTT and 4-MSOB HTT in collected urine within 24 h compared to the amount of intake was 18 ± 5% for allyl HTT and 21 ± 4% for 4-MSOB HTT.

#### Stability of 4-Hydroxythiazolidine-2-Thiones in a Stomach Model

In an *in vitro* stomach model, the allyl HTT and 4-MSOB HTT showed no degradation during the 2 h incubation at 37°C in the stomach model solution at pH 2 compared to incubation in pure water at room temperature. The recoveries were 98 ± 2% for allyl HTT and 98 ± 3% for 4-MSOB HTT compared to the pure water control reference.

#### Uptake of 4-Hydroxythiazolidine-2-Thiones Into HepG2 Cells

In addition, the uptake of both HTTs into cancer cells was evaluated. To avoid false-positive results due to residues of allyl HTT and 4-MSOB HTT from the treatment solution, cells were washed and the analyte amount in the cell supernatant before cell lysis was estimated by quantification of the concentration and estimation of the volume. It was assumed that the increase of analyte amount during cell lysis originated from analytes located inside the cells. The results are shown in Table 2. The amount of both HTTs increased significantly (*t*-test, *p* = 0.02 for allyl HTT, and *p* = 0.003 for 4-MSOB HTT using six washing steps) by cell lysis, indicating that allyl HTT and 4-MSOB HTT were taken up by HepG2 cells. For 4-MSOB HTT, no significant increase due to cell lysis was obtained in the first approach using only three washing steps, based on the high amount of analyte residue from the former incubation compared to 4-MSOB HTT levels released from cells. Therefore, the number of washing steps was increased to six, resulting in a detectable increase of 4-MSOB HTT levels due to cell lysis. The increased amount of analyte in the supernatant, due to cell lysis, was clearly higher for allyl HTT, compared to 4-MSOB HTT. It must be considered that uptaken HTTs could have been metabolized, which would reduce HTT levels in the cells. So the determined mounts represent the minimum of cellular uptaken HTT amounts.

**Table 2:** Minimum cellular uptake of 3-allyl-4-hydroxythiazolidine-2-thione (allyl HTT) and 4-hydroxy-3-(4-(methylsulfinyl)butyl)thiazolidine-2-thione (4-MSOB HTT) into HepG2 cells as measured by LC-MS/MS as content in the supernatant before and after cell lysis with estimation of volumes of the supernatants before ultrasonic and acetonitrile treatment (*n* = 3 biological replicates, data shown as mean ± SD).

**Analyte**	**Amount of analyte in supernatant before cell lysis**	**Amount of analyte in supernatant after cell lysis**	**Increase due to cell lysis**
Allyl HTT (3 washing steps)	520 ± 170 fmol	1,060 ± 140 fmol	540 ± 160 fmol
4-MSOB HTT (3 washing steps)	760 ± 190 fmol	810 ± 140 fmol	56 ± 55 fmol
4-MSOB HTT (6 washing steps)	1.7 ± 0.3 fmol	21 ± 5 fmol	20 ± 5 fmol

Additionally, medium spiked with allyl HTT or 4-MSOB HTT was measured after 24 h of treatment to check for newly formed metabolites by the HepG2 human cancer cells. For allyl HTT, the measurements by HPLC-UV did not show any additional signals in the LC-UV chromatogram, compared to the spiked medium which was not in contact with HepG2 cells. However, in the case of 4-MSOB HTT, a new peak was observed, which was not detectable in spiked medium without 24 h of cell incubation and not detectable in non-spiked medium incubated 24 h with HepG2 cells (Supplementary Figure S2). The retention time of the new peak at 10.1 min indicates that the resulting metabolite is less polar compared to 4-MSOB HTT with a retention time of 7.6 min. The UV spectra of 4-MSOB HTT and the transformation product were very similar with a maximum at 274 nm, which supports the assumption that it is a transformation product of 4-MSOB HTT. The relative signal intensity of the transformation product compared to 4-MSOB HTT in a 24 h incubated medium in presence of cells was 0.3% of the 4-MSOB HTT signal. To characterize the transformation product, the samples were measured by HPLC-DAD-ESI-qToF. Based on the high-resolution mass spectra (Supplementary Figure S3), a possible composition for the metabolite with C_16_H_30_N_2_O_2_S_6_ measured as C_16_H_30_N_2_O_2_S_6_-Cu^+^ *m/z* 536.9924 (calculated 536.9922, difference 0.4 ppm) was obtained. The molecular formula indicates that it is formally a dimer of the reduced form of 4-MSOB HTT. However, a simple metal ion dimer complex is unlikely, because the corresponding K^+^ and Na^+^ adducts were observed, as well. In Supplementary Figure S4, the extracted ion chromatograms (EICs) obtained from the LC-MS measurement, using the full scan mode, are shown. Besides different adducts, such as Cu^+^, K^+^, and Na^+^ adducts, insource oxidation, and insource demethylation (loss of CH_3_, as an insource fragmentation), were observed for the formed metabolite of 4-MSOB HTT (retention time: 10.9 min). Oxidation of the thioether group (reduced 4-MSOB HTT) and fragmentation involving neutral loss of CH_3_ (demethylation of the S-CH_3_) are expectable reactions under insource ESI ionization conditions. Additionally, the formation of 4-MSOB HTT during insource fragmentation of the reduced 4-MSOB HTT dimer metabolite is visible, whereby the Na^+^ adduct is the predominant adduct species, such as for 4-MSOB HTT (retention time: 7.8 min). The suggestion of a reduced dimer of 4-MSOB HTT was supported by the fact that UV spectra of 4-MSOB HTT and the new metabolite were comparable, the fact that the reduced 4-MSOB HTT dimer would be less polar compared to 4-MSOB HTT (as observed with later retention time), and that 4-MSOB HTT is formed out of the new metabolite by insourcing fragmentation (and insource oxidation) during mass spectrometry measurements. Moreover, allyl HTT, for which no transformation product was observed, would not be able to form an analog transformation product because of the different site chain.

#### Transport of 4-Hydroxythiazolidine-2-Thiones Through *in vitro* Model of the Intestinal Barrier

The potential of allyl HTT and 4-MSOB HTT to overcome the intestinal barrier was evaluated by using a model of a monolayer of differentiated Caco-2 cells. The transport through the layer was tested by quantification of analyte concentrations in the compartment which was spiked with the analyte and the compartment on the opposite side of the layer over time. The results are shown in Table 3. Results show that the equilibrium of a concentration ratio of 1:1 between the upper and lower compartment is achieved more quickly for allyl HTT than for 4-MSOB HTT. Additionally, the enhanced transport of allyl HTT is noticeable by the fact that after 2 min allyl HTT is detectable in the opposite compartment but not 4-MSOB HTT. In general, it seems that in the inverse exposure experiment (analyte spiked in the lower compartment), the transport was faster compared to the first experiment. In the inverse exposure experiment after an incubation time of 24 h, the concentration equilibrium for 4-MSOB HTT was reached, in contrast to the first experiment, in which a ratio of approximately 2:1 was obtained after 24 h incubation. It should be noted that the volumes of analyte spiked medium were different for the different experiments (2 vs. 2.7 mL to achieve a hydrodynamic equilibrium in both experiments), and, therefore, the spiked amount of analyte was different with 35 μg for the first experiment and 47 μg for the inverse exposure experiment (Supplementary Figure S1) that could affect reaching the equilibrium of concentrations in the two compartments, which is approximately 40 μM for the first approach and approximately 60 μM for the inverse exposure experiment. The integrity of the cellular barriers was tested by TEER measurements with net resistance values (measured resistance of cell-loaded system minus measured resistance of cell-free system but with transwell insert and medium) of 130–180 Ω after 3 days proliferation time, 225–285 Ω after 1 week, 270–350 Ω after 2 weeks, 290–360 Ω after 3 weeks, and 285–370 Ω after treatment with analytes and sampling of aliquots for HPLC-UV measurements. The resistance values indicate that the integrity of the layer was nearly achieved after 2 weeks of proliferation time and the layer was neither disturbed by incubation with analytes nor by a sampling of medium samples from the different compartments.

**Table 3:** Transport of 3-allyl-4-hydroxythiazolidine-2-thione (allyl HTT) and 4-hydroxy-3-(4-(methylsulfinyl)butyl)thiazolidine-2-thione (4-MSOB HTT) through an *in vitro* model of the intestinal barrier by spiking the compound in the upper compartment (transwell insert) or lower compartment (well) to a closed Caco-2 cell layer and sampling from both compartments after different incubation times (*n* = 6 biological replicates, results shown as mean ± SD).

**Incubation time after spiking**	**Compound was spiked in the**				**Inverse exposure: compound**			
	**upper compartment**				**was spiked in the lower compartment**			
	**2 min**	**1 h**	**2 h**	**24 h**	**2 min**	**1 h**	**2 h**	**24 h**
**Concentrations of allyl HTT in** **μM**								
Upper compartment	100 ± 2	81 ± 2	61 ± 2	36 ± 1	0.2 ± 0.1	37 ± 4	56 ± 7	65 ± 2
Lower compartment	0.6 ± 0.3	14 ± 2	26 ± 3	35 ± 0	105 ± 8	94 ± 8	88 ± 8	64 ± 2
Concentration ratio of compartment non-spiked vs. spiked in %	1	17	42	96	0	39	64	102
**Concentrations of 4-MSOB HTT in** **μM**								
Upper compartment	112 ± 9	97 ± 6	99 ± 3	47 ± 1	0.0 ± 0.0	5 ± 1	11 ± 2	65 ± 4
Lower compartment	0.0 ± 0.0	0.8 ± 0.5	2.7 ± 0.4	25 ± 6	106 ± 5	103 ± 4	98 ± 13	65 ± 2
Concentration ratio of compartment non-spiked vs. spiked in %	0	1	3	54	0	5	11	99

#### Determination of Log P (Partition Coefficient in Octanol/Water) Values of 4-Hydroxythiazolidine-2-Thiones

The concentration ratio of allyl HTT in the octanol phase compared to the water phase was 8.1 ± 0.4, which corresponds to a log *P*-value of 0.91 ± 0.02. For the more polar 4-MSOB HTT, a concentration ratio between the octanol phase and water phase of 0.45 ± 0.04 was determined, which corresponds to a log *P*-value of −0.35 ± 0.04. The log *P*-value is positive for compounds which are better soluble in the octanol phase, and is negative for compounds which are better soluble in the aqueous phase. To assess if the different uptake behavior of the two HTTs is based on their different polarity, the octanol/water partition coefficients of several compounds were plotted against their permeability coefficients, based on a previous report by Levin (32) (Supplementary Figure S5). The obtained plot was used to estimate the permeability coefficients of the two HTTs with 2 × 10^−4^ cm/s for allyl HTT and 1.1 × 10^−5^ cm/s for 4-MSOB HTT using the measured octanol/water partition coefficients of both compounds. The ratio of both estimated permeability coefficients was 18 and fitted very well to the measured ratios of 27-fold and 10-fold for the higher uptake of allyl HTT compared to 4-MSOB HTT in the experiments with HepG2 and Caco-2 cells. Therefore, the uptake of allyl HTT and 4-MSOB HTT in the *in vitro* assays can be primarily explained by simple passive diffusion through the cell membrane. Additionally, the fact that in both intestinal model experiments (transport of the analyte from the upper to the lower compartment, as well as from the lower to an upper compartment), the transport velocity was similar, and an equilibrium concentration between both compartments was reached, supporting the hypothesis of passive diffusion without the need of special transporters.

### Bioactivity of 4-Hydroxythiazolidine-2-Thiones

#### Determination of Cytotoxic Effects of 4-Hydroxythiazolidine-2-Thiones

**Figure 2:**
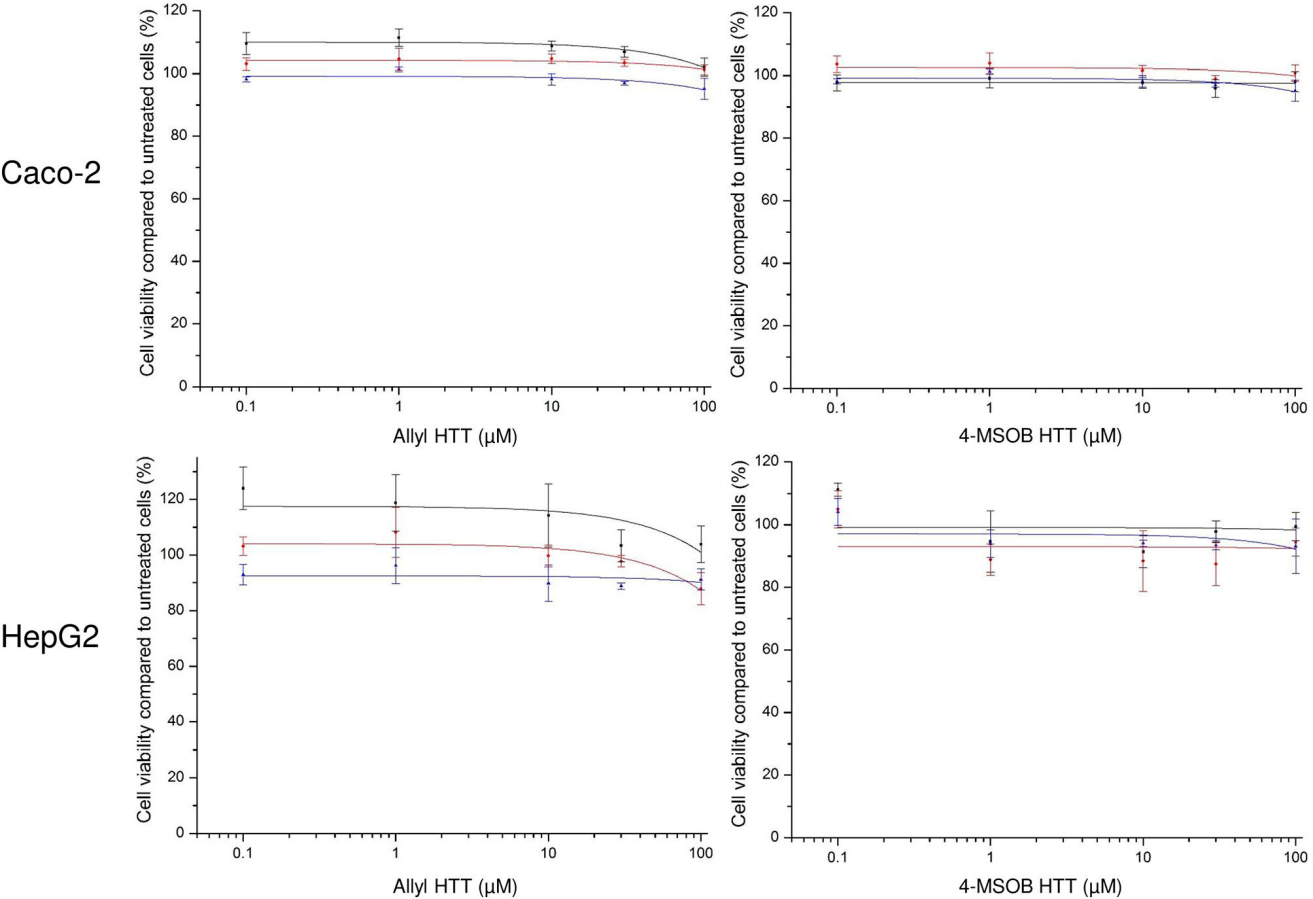
Effect of 3-allyl-4-hydroxythiazolidine-2-thione (allyl HTT, left) and 4-hydroxy-3-(4-(methylsulfinyl)butyl)thiazolidine-2-thione (4-MSOB HTT, right) on cell viability of Caco-2 cells (above) or HepG2 cells (below) measured by neutral red uptake assay. Depicted as ratio between measured absorbance values for cells treated with analyte spiked medium vs. treated with non-spiked medium. Each data point represents mean ± SD of three technical replicates and each colored line represents an independent cell passage of the 3 biological replicates.

The incubation of allyl HTT and 4-MSOB HTT at concentration levels up to 100 μM did not show any effect on cell viability compared to untreated HepG2 or Caco-2 cells, using the neutral red uptake assay (Figure 2) or the MTT assay (Supplementary Figure S6).

#### Determination of Antioxidant Potential of 4-Hydroxythiazolidine-2-Thiones

For determination of the antioxidant potential of allyl HTT and 4-MSOB HTT, ABTS and DPPH were used to form stable radicals, which can be eliminated by radical scavengers (antioxidants). Trolox was used as a positive control. In the ABTS assay, the addition of 100 μM Trolox solution as an antioxidant led to a reduction of the radical signal of 31.2 ± 0.4% compared to adding a blank solution. A 6.25 μM Trolox solution reduced the radical signals by 1.3 ± 0.4% in the ABTS assay and 0.8 ± 0.3% in the DPPH assay. In the case of allyl HTT and 4-MSOB HTT, no reduction of the extinction value of the radicals was observed in the ABTS assay or DPPH assay up to 100 μM. The detailed results are shown in Supplementary Table S1.

#### Determination of Glycogen Synthase Kinases-3 Inhibition by 4-Hydroxythiazolidine-2-Thiones

**Figure 3:**
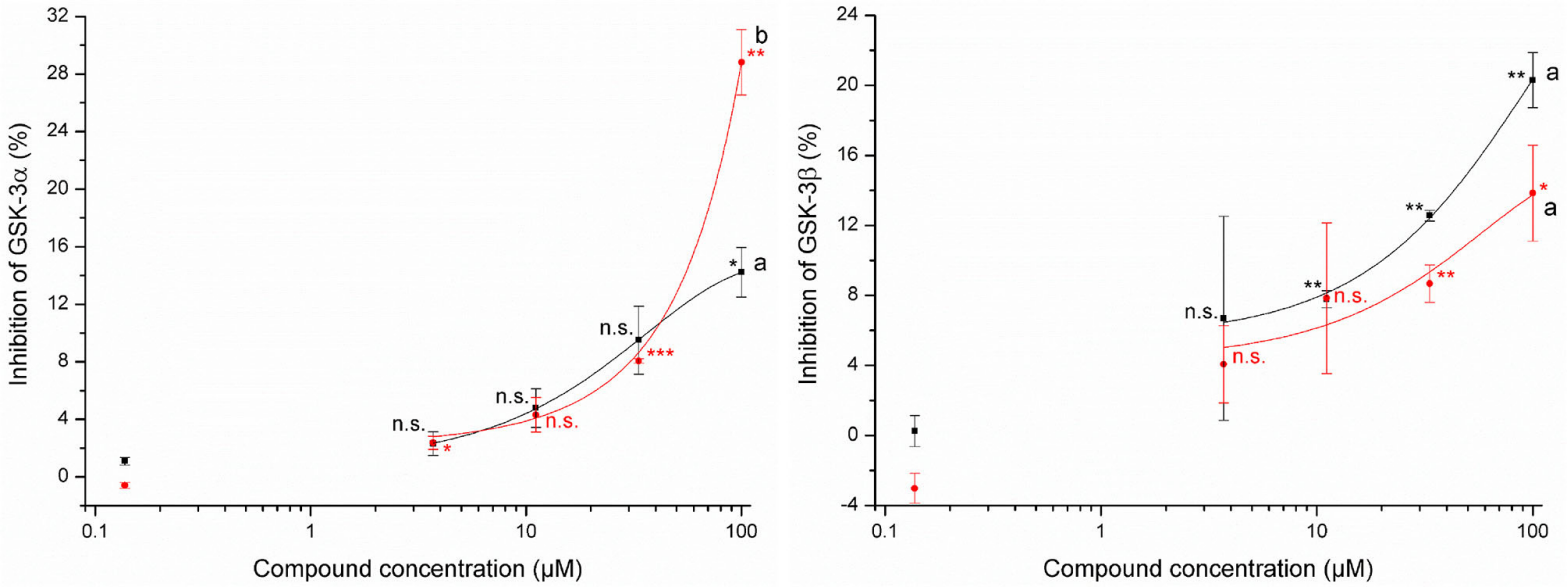
Effects of 3-allyl-4-hydroxythiazolidine-2-thione (allyl HTT, black square) and 4-hydroxy-3-(4-(methylsulfinyl)butyl)thiazolidine-2-thione (4-MSOB HTT, red circle) on the inhibition of glycogen synthase kinases-3α (left) and glycogen synthase kinases-3β (right) by using Z'-LYTE assay (*n* = 2, data shown as mean ± SD) with different letters for significant different results between both HTTs at 100 μM (*t*-test, *p* < 0.05). Stars report significant differences of means as tested by *t*-test of the concentration level compared to the standard with 0.14 μM at different significance levels: *p ≤ 0.05, **p ≤ 0.01, ***p ≤ 0.001, and n.s. (not significant, *p* > 0.05).

Tested concentrations of up to 100 μM showed a slight potential of allyl HTT and 4-MSOB HTT to inhibit glycogen synthase kinases-3 (Figure 3). Significant inhibition (*p* ≤ 0.05) of both GSK-3 isoforms by both HTTs compared to the lowest tested HTT concentration of 0.14 μM was only obtained for 100 μM of the tested HTTs. At this level, a significantly different inhibition (*t*-test, *p* = 0.036) by 4-MSOB HTT compared to allyl HTT was obtained for GSK-3α, whereby no significant difference in inhibition between both HTTs was found for the isoform GSK-3β (*t*-test, *p* = 0.10). Higher levels of allyl HTT result in an inhibition of GSK-3β with greater statistical significance (*p* ≤ 0.01) in contrast to 4-MSOB HTT, which leads to an inhibition of GSK-3α (*p* ≤ 0.01). Based on the results of the Z'-LYTE assay, a conclusion on which kind of inhibition (competitive or non-competitive) occurs is not possible since the assay only determines the reduction in kinase activity in general.

#### Determination of Changes in NAD(P)H Dehydrogenase Quinone 1 Activity by 4-Hydroxythiazolidine-2-Thiones

The used HepG2 cells showed a high NQO1 activity of 145 ± 8 mU/mg protein even in the negative control. The positive control, using auranofin, led to a measured activity of 153 ± 4 mU/mg protein. Due to this insufficient increase in activity by chemical induction, HCT116 pTRAF was tested. The positive control 2 μM auranofin showed an NQO1 activity of 114 ± 7 mU/mg protein, which was significantly (*t*-test, *p* = 0.0003) increased compared to the negative control (51 ± 5 mU/mg protein). Both HTTs led to NQO1 activities in the range of 49 ± 2 mU/mg protein to 52 ± 2 mU/mg protein, and, thereby, did not show any significant changes in NQO1 activity, compared to the negative control. No significant differences were obtained for the tested concentrations of 1 μM and 100 μM HTT.

## Discussion

Cabbages, such as red cabbage, are often consumed after boiling, which leads to the formation of HTTs as products from ITC and mercaptoacetaldehyde derived from the GLS degradation product thioglucose (20). In the present study, after consumption of red cabbage broth, the thermal GLS degradation products (allyl HTT and 4-MSOB HTT) were recovered in human urine to 18 ± 5% and 21 ± 4% within 24 h. This demonstrates the uptake of these substances in the human circulatory system. 4-MSOB HTT is formed during food preparation by the reaction of the ITC sulforaphane and mercaptoacetaldehyde (20). The bioavailability of sulforaphane was reported to be approximately 40% (33) and is therefore comparably higher than of 4-MSOB HTT. The corresponding ITC to allyl HTT is allyl ITC. The recovery of formed allyl ITC metabolites in 10–12 h urine after consumption of horseradish or a mustard paste was in the range of 42–54% (34, 35). Mainly, the *N-*acetylcysteine conjugates were detected in urine as it was described for benzyl ITC as well (36). The highly reactive ITCs are conjugated with glutathione, followed by several other enzymatic modifications of the mercapturic acid pathway. In contrast, the thermal GLS degradation products, allyl HTT and 4-MSOB HTT, are not as reactive as ITCs. Moreover, they were detected non-metabolized in urine. After *in vitro* incubation of the HTTs with HepG2 cells, only for 4-MSOB HTT, a small additional peak was detectable in the LC-UV measurements, which indicates that a new metabolized product was formed. Using high-resolution mass spectrometry, a dimer of the reduced form of 4-MSOB HTT can be suggested as this new metabolite. However, based on the apparently low level of the product we tentatively suggest that the detected *in vitro* metabolite plays just a minor role *in vivo* although the recoveries of approximately 20% for 4-MSOB HTT in urine were comparatively low. It must be noted that, for determination of the recoveries of allyl HTT and 4-MSOB HTT, red cabbage was consumed without additives. The addition of fat might increase the uptake of allyl HTT and 4-MSOB HTT, as was shown in an *in vivo* study, where consumption of a whole meal that included meat, the absorption of ITC was between 1- and 5-fold higher compared to ingestion without meat depending on the type of ITC (37). This effect was explained by the presence of fat that can increase the absorption of lipophilic compounds through the micellar phase (38). The bioavailability for allyl HTT and 4-MSOB HTT was ~20%. This raises the question of incomplete uptake or incomplete metabolization. Reasons for the comparable low recoveries could be as follows: (1) the metabolization to unknown metabolites, which were not formed by the tested cell lines, but in humans or by the microbiome, (2) conjugation with proteins, (3) immobilization in cells, and (4) no uptake and excretion *via* feces. In further studies, analyses of blood and feces samples could help to answer the question of the whereabouts of the remaining portion of HTTs. To evaluate the potential of uptake of allyl HTT and 4-MSOB HTT, several models were used. Both compounds were stable in an *in vitro* stomach model experiment. The potential of a substance to be taken up by passive diffusion can be estimated by its log *P*-value. For optimal intestinal absorption, a log *P* range of 0.5–2 is suggested (21). The determined log *P*-values of allyl HTT and 4-MSOB HTT were 0.91 and −0.35 and were therefore within or near the optimal log *P* range for passive diffusion through cell membranes. Based on the experiment using HepG2 cells (27-fold) and Caco-2 cells (10-fold), the uptake of allyl HTT was clearly higher compared to 4-MSOB HTT, and the findings agree with estimated permeability coefficients based on the determined log *P*-values of both HTTs, which indicate that the uptake of the HTTs is due by simple passive diffusion.

The uptake of the tested HTTs raises the question of their bioactivity. The tested allyl HTT and 4-MSOB HTT did not show any acute cytotoxicity in HepG2 and Caco-2 cells of up to 100 μM, which is at least 100-fold higher than expected physiological concentrations after an ordinary meal. In contrast, the cytotoxicity of the corresponding ITCs was reported much higher. Sulforaphane induced apoptosis and cytotoxicity at concentrations of 20–40 μM in endothelial cells (39), but while in a range of 10 μM and 50 μM, no significant cytotoxicity on cultured hepatocytes from human liver transplants was determined for sulforaphane (40). Using the cancer cell line HepG2 after 72 h of incubation time, IC_50_ (50% inhibition of cell growth) values of 36 μM for allyl ITC and 13 μM for sulforaphane were reported (41). In contrast to the reactive ITCs, for *N*-methyl-2-thiazolidinethione, a structurally similar motif to allyl HTT and 4-MSOB HTT, low toxicity was described (42). In addition to no toxicity, health-promoting properties of allyl HTT and 4-MSOB HTT would be desirable. For both compounds, no antioxidant potential was detected in the present study, probably due to the lack of redox-active functional groups. Additionally, the potential to inhibit GSK-3 by allyl HTT and 4-MSOB HTT was tested, based on the results of Noori et al. (24), who reported a potential to inhibit GSK-3 for a thione called COB-187, structurally similar to allyl HTT and 4-MSOB HTT. COB-187 showed IC_50_ (50% inhibition) values of 0.022 μM to inhibit isoform GSK-3α and 0.011 μM to inhibit GSK-3β. The HTTs showed inhibition of GSK-3α and GSK-3β, however only 29% at maximum on a concentration of 100 μM. At concentration levels of 1 μM, which is in a realistic range based on determining amounts of allyl HTT and 4-MSOB HTT in boiled red cabbage, no relevant inhibition of GSK-3 can be assumed. However, the potential to inhibit GSK-3 was evident and a reduction of GSK-3β activity can be related to an increase in the Nrf2 activity (43). Due to the fact that NQO1 is related to Nrf2 (44), NQO1 activity was measured to investigate indirectly Nrf2 activity. Both HTTs showed no effects on NQO1 activity up to 100 μM. Regarding the Nrf2 pathway, Wise et al. found no stimulation in Nrf2 target gene expression in alveolar macrophages and bronchial epithelial cells after intervention with 150 μM sulforaphane as well (45).

The recently discovered GLS transformation products did not show evident bioactivity at expected physiological concentrations in the limited number of tested assays of the present study. Additional assays could be used to test the potential of HTTs as antidiabetic drugs based on the potential of structurally similar compounds, and several additional bioactivities are reported for different thiazolidinones as well (22). Although HTTs have shown no negative bioeffects in the tested assays, their abundance is associated with the depletion of beneficial ITCs, which are precursors of HTTs. Based on the presented limited bioactivity experiments, the formation of HTTs should be avoided to not reduce ITC levels. We have shown recently that the formation of HTTs is strongly reduced under acidic conditions (pH 6) compared to neutral conditions (20). Additionally, acidic conditions and shorter boiling times affect ITC levels to a lesser extent (46). Thus, if red cabbage is boiled during food preparation, using vinegar and short boiling times are recommended to best maintain ITC levels.

## Conclusion

In the present study, it was shown that the recently discovered thermal GLS degradation products 3-allyl-4-hydroxythiazolidine-2-thione and 4-hydroxy-3-(4-(methylsulfinyl)butyl)thiazolidine-2-thione are taken up when consuming boiled vegetables or water used for the boiling. This was reflected in the recovery of the two compounds of approximately 20% in collected 24 h urine after ingestion of cabbage cooking water. Both HTTs can overcome the stomach and intestinal barrier in *in vitro* model experiments, and passive diffusion seems to be the most likely uptake mechanism. Although taken up, no apparent biological effects were revealed in the tested assays including in this study, suggesting that HTTs may not contribute to health effects related to GLS consumption. The compounds were not bioactive in concentration levels relevant for foods in any of the four used assays. Further characterization by additional assays is needed to obtain a more profound impression of their contribution to human nutrition.

## Data Availability Statement

The raw data supporting the conclusions of this article will be made available by the authors, without undue reservation.

## Ethics Statement

Ethical review and approval was not required for the study on human participants in accordance with the local legislation and institutional requirements. The patients/participants provided their written informed consent to participate in this study.

## Author Contributions

HH was responsible for the visualization, investigation, and writing the original draft. CO, JR, LA, MR, TG, and FH were involved in reviewing and editing the original draft. LA and MR were involved in practical work. HH, CO, JR, TG, and FH contributed to the study conceptualization and methodology. FH was involved in project administration and funding acquisition. All authors contributed to the writing.

## Funding

This study was funded by Leibniz Association (Leibniz Junior Research Group OPTIGLUP; J16 / 2017).

## Conflict of Interest

The authors declare that the research was conducted in the absence of any commercial or financial relationships that could be construed as a potential conflict of interest.

## Publisher's Note

All claims expressed in this article are solely those of the authors and do not necessarily represent those of their affiliated organizations, or those of the publisher, the editors and the reviewers. Any product that may be evaluated in this article, or claim that may be made by its manufacturer, is not guaranteed or endorsed by the publisher.
