# Supplementary material for: Assessing Bioavailability and Bioactivity of 4-Hydroxythiazolidine-2-Thiones, Newly Discovered Glucosinolate Degradation Products Formed During Domestic Boiling of Cabbage
Source: Front Nutr. 2022 Jul 22;9:941286. doi: 10.3389/fnut.2022.941286 (PMC9354954; doi:10.3389/fnut.2022.941286)
Supplement: Supplementary file 1 [file Data_Sheet_1.DOCX]

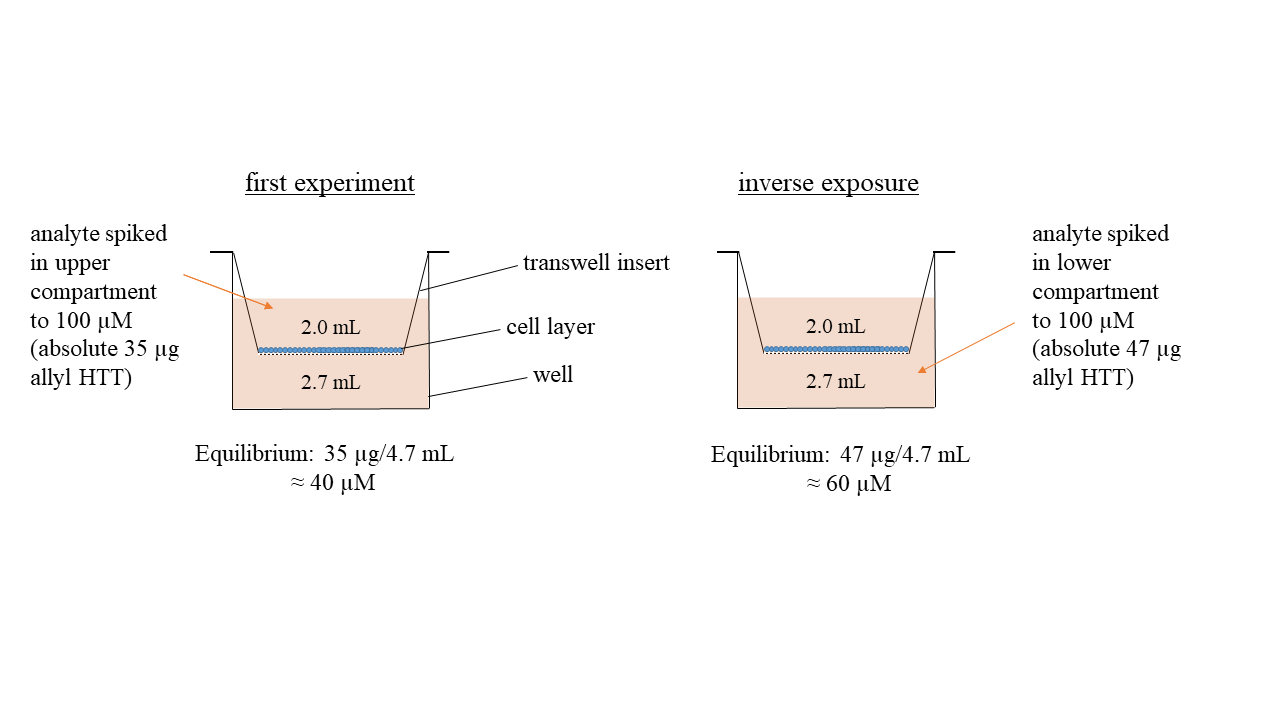


Figure S1: Spiking scheme of 4-hydroxythiazolidine-2-thiones (HTTs) in medium to evaluate their transport in an *in vitro* model of the intestinal barrier.


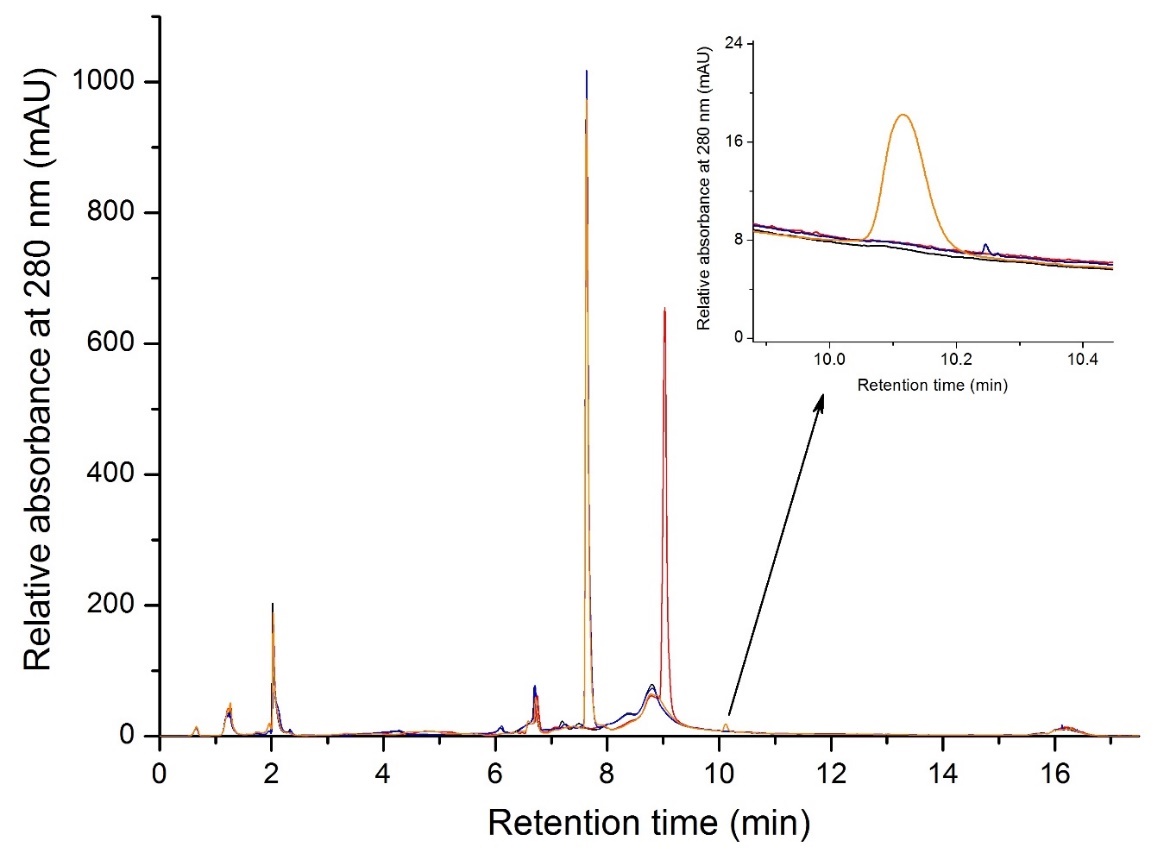


Figure S2: LC-UV Chromatogram of samples treated for 24 h under cell culture conditions using either (black line) unspiked medium in the presence of HepG2 cells, (red line) medium spiked with 100 µM 3-allyl-4-hydroxythiazolidine-2-thione (allyl HTT) in the presence of HepG2 cells, (orange line) medium spiked with 100 µM 4-hydroxy-3-(4-(methylsulfinyl) butyl)thiazolidine-2-thione (4-MSOB HTT) in presence of HepG2 cells, and (blue line) medium spiked with 100 µM 4-MSOB HTT in absence of HepG2 cells.

Figure S3: High resolution mass spectrum (ESI positive) obtained by using HPLC-qToF of a transformation product of 4-hydroxy-3-(4-(methylsulfinyl)butyl)thiazolidine-2-thione (4‑MSOB HTT) after 24 h incubation of 4-MSOB HTT with HepG2 cells, shown with the expected isotope distribution indicated by the red rectangles (expected isotope signal intensity is shown by the height of the red rectangles and the expected masses by the position of the red rectangles) for molecular formula C_16_H_30_N_2_O_2_S_6_-Cu^+^.


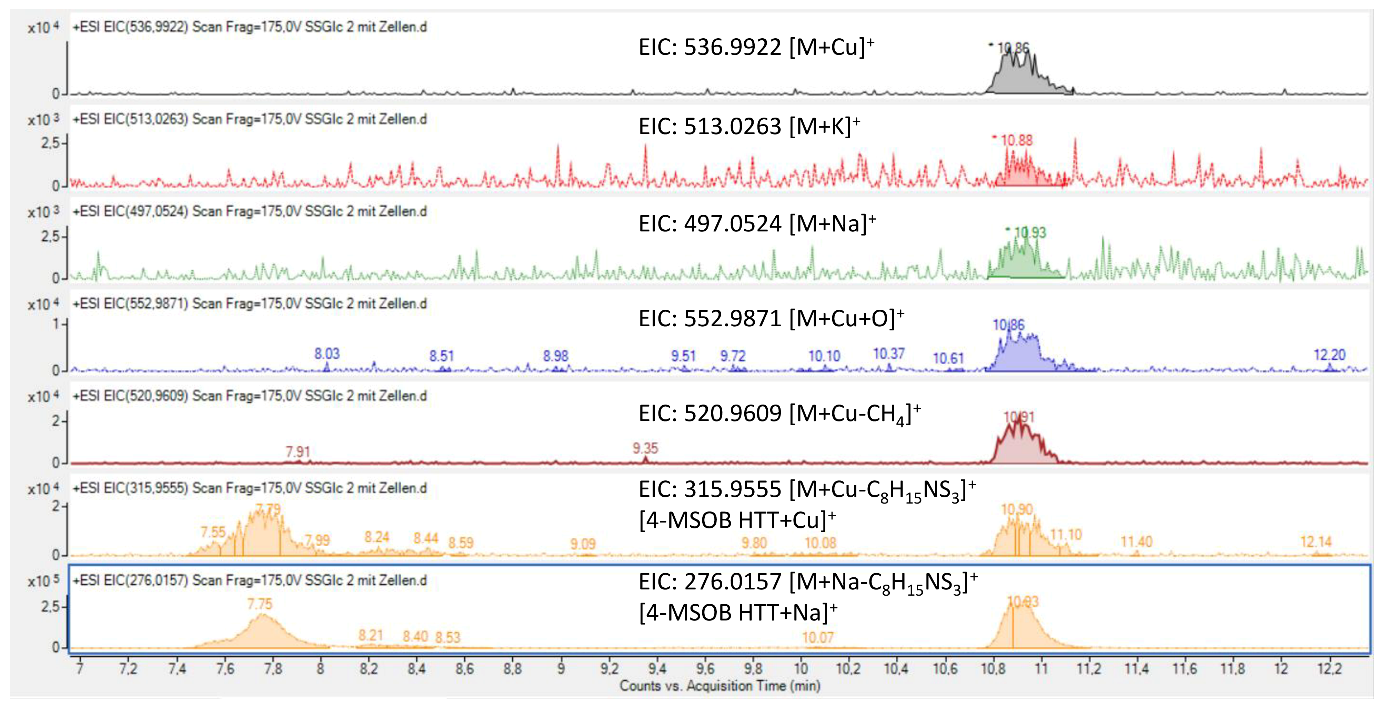


Figure S4: Extracted ion chromatograms (EICs) obtained from the LC-HRMS full scan measurement, shown for the expected *m/z* of the Cu^+^, K^+^, and Na^+^ adduct of the potential 4‑MSOB HTT transformation product (C_16_H_30_N_2_O_2_S_6_, reduced 4‑MSOB HTT dimer) as well as for the product formed by insource oxidation during ionization, insource demethylation fragmentation (-CH_4_) and insource fragmentation to 4-MSOB HTT (‑C_8_H_15_NS_3_) shown as Cu^+^ and Na^+^ adduct at 10.9 min for the potential reduced 4‑MSOB HTT dimer. The retention time of 4-MSOB HTT is 7.8 min.


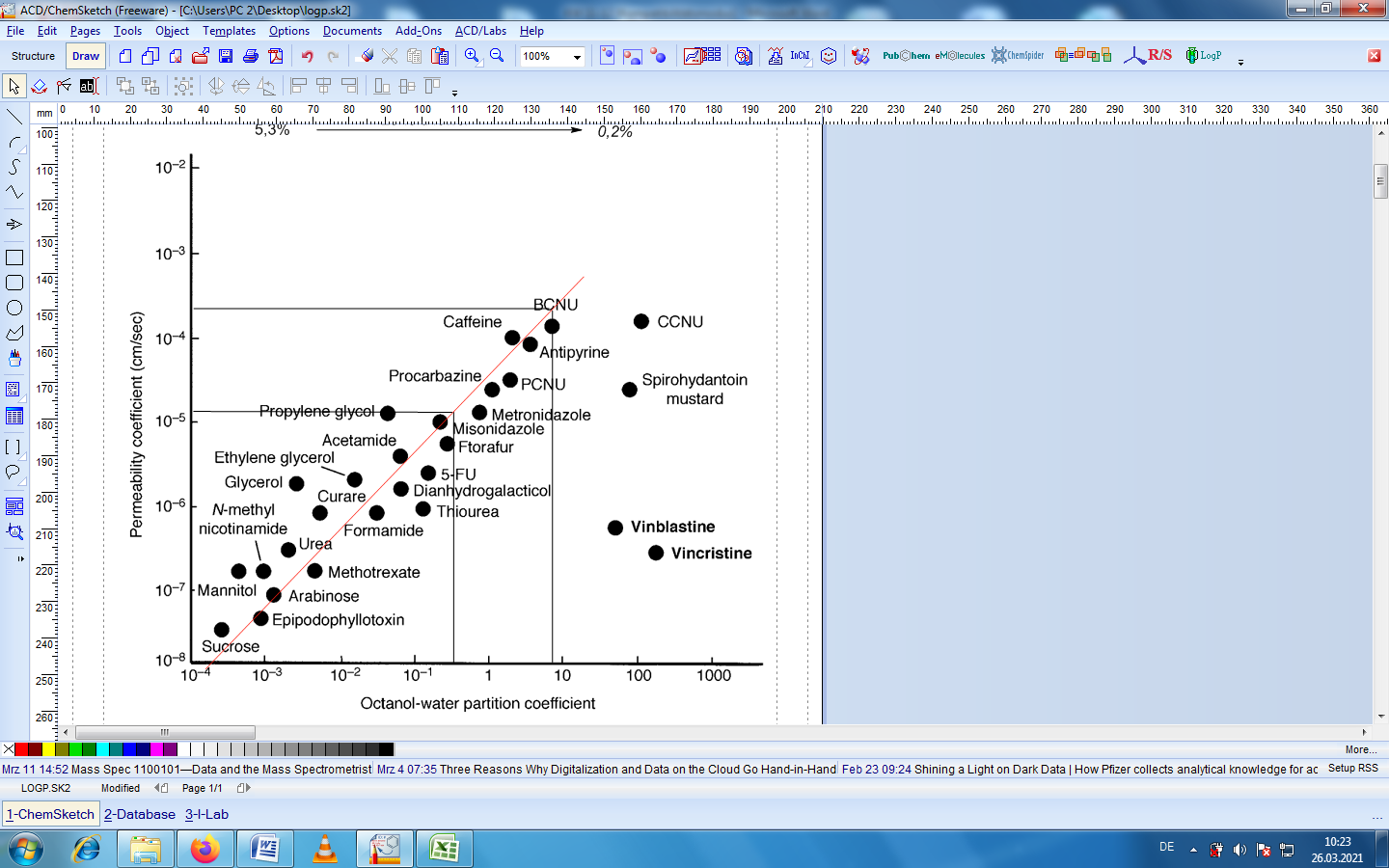


Figure S5: Plot of data reported by Levin (1980) to describe the correlation between the octanol/water partition coefficients of several compounds and their permeability coefficients. The red line and thin black lines were draw by the author and used to estimate the permeability coefficients of 3‑allyl-4-hydroxythiazolidine-2-thione (allyl HTT) and 4-hydroxy-3-(4-(methylsulfinyl)butyl) thiazolidine-2-thione (4-MSOB HTT) using their measured octanol-water partition coefficients (black lines).


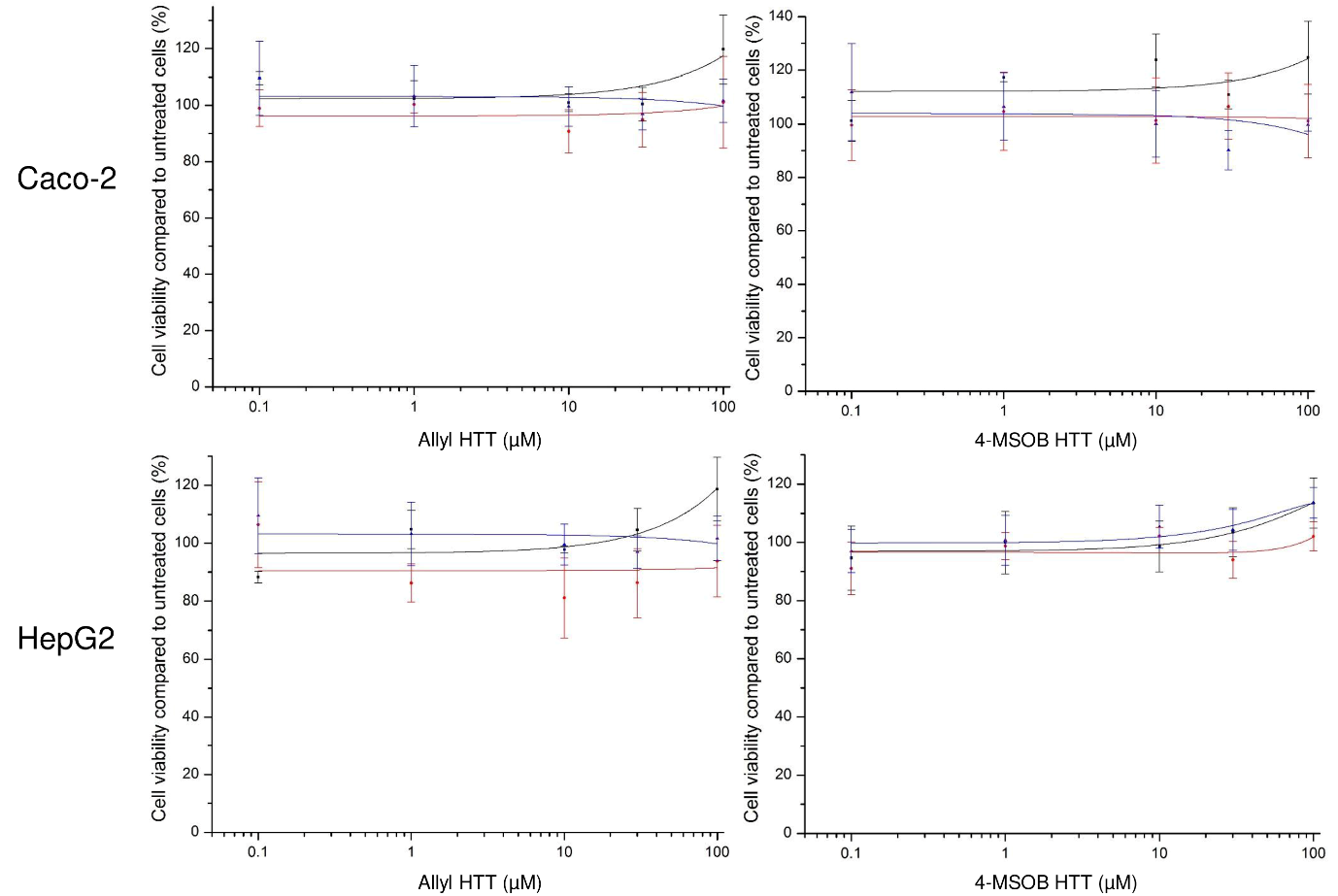


Figure S6: Effect of 3-allyl-4-hydroxythiazolidine-2-thione (allyl HTT, left) and 4‑hydroxy-3-(4-(methylsulfinyl)butyl)thiazolidine-2-thione (4-MSOB HTT, right) on cell viability of Caco‑2 cells (above) or HepG2 cells (below) measured by MTT assay. Depicted as ratio between measured absorbance values for cells treated with compound spiked medium versus treated with non-spiked medium. Each data point represents the mean ± SD of 3 technical replicates and each colored line represents an independent cell passage (3 biological replicates).

Table S1: Relative signal reduction in % of the extinction value of the radicals ABTS**^ꞏ^** or DPPH**^ꞏ^** using different concentrations of Trolox (positive control), 3-allyl-4-hydroxythiazolidine-2-thione (allyl HTT) and 4-hydroxy-3-(4-(methylsulfinyl)butyl)thiazolidine-2-thione (4‑MSOB HTT) compared to a compound free blank sample to determine the antioxidant potential

| Concentration (µM) | ABTS assay | | | DPPH assay | | |
| --- | --- | --- | --- | --- | --- | --- |
|  | Trolox | Allyl HTT | 4-MSOB HTT | Trolox | Allyl HTT | 4-MSOB HTT |
| 6.25 | 1.3 ± 0.4 | -0.2 ± 0.3 | 0.6 ± 0.5 | 0.8 ± 0.3 | 0.2 ± 0.3 | 0.3 ± 0.4 |
| 12.5 | 1.9 ± 0.4 | -0.3 ± 0.3 | -0.1 ± 0.5 | 2.8 ± 0.3 | 0.0 ± 0.3 | 0.0 ± 0.3 |
| 25 | 9.3 ± 0.5 | 0.4 ± 0.4 | -0.5 ± 0.4 | 3.5 ± 0.4 | 0.1 ± 0.3 | 0.3 ± 0.3 |
| 50 | 16.5 ± 0.4 | 0.3 ± 0.5 | 1.1 ± 0.8 | 8.8 ± 0.6 | 0.1 ± 0.3 | 0.3 ± 0.4 |
| 100 | 31.2 ± 0.4 | -0.3 ± 0.3 | 0.4 ± 0.5 | 17.5 ± 1.5 | 0.0 ± 0.3 | 0.3 ± 0.4 |
